# Evaluation of radiological instability signs in the distal radioulnar joint in children and adolescents with arthroscopically-verified TFCC tears

**DOI:** 10.1007/s00402-020-03470-y

**Published:** 2020-05-07

**Authors:** Florian Schachinger, Sascha Wiener, Marcos F. Carvalho, Michael Weber, Rudolf Ganger, Sebastian Farr

**Affiliations:** 1 Department of Pediatric Orthopaedics and Foot and Ankle Surgery, Orthopedic Hospital Speising, Medical University Vienna, Speisingerstrasse 109, 1130 Vienna, Austria; 2 II. Orthopedic Department, Herz-Jesu Hospital, Vienna, Austria; 3 EPE, Department of Pediatric Orthopaedics, Pediatric Hospital of Coimbra, CHUC, Coimbra, Portugal; 4 Departement of Biomedical Imaging and Image-Guided Therapy, Medical University Vienna, Vienna, Austria

**Keywords:** DRUJ, Instability, TFCC tear, Triangular fibrocartilage complex, Pisoscaphoid distance, Radioulnar distance, Radioulnar ratio, Wrist

## Abstract

**Introduction:**

Recent reports in the adult literature reported the use of standardized radiographic measurement techniques to determine distal radioulnar joint (DRUJ) instability. The aim of this study was to evaluate the efficacy and accuracy of (1) the MRI-based modified radioulnar ratio technique and (2) the pisoscaphoid (PiSca) and radioulnar (RaUl) distances in true lateral radiographs in children and adolescents with arthroscopically-verified TFCC tears.

**Materials and methods:**

We retrospectively assessed lateral wrist radiographs and axial MRI sequences of 18 adolescent patients (22 wrists) who had arthroscopically-confirmed TFCC tears and compared them to similar imaging of a control group of 28 healthy patients (28 wrists). Three raters assessed the images twice in a 2-week interval. Intraclass correlation coefficients (ICCs), unifactorial ANOVA, and ROC analysis were performed with regards to the different radiographic variables.

**Results:**

The interrater ICCs were almost perfect for all measurements except RaUl1, which showed a substantial agreement (0.751) among the three observers. The intrarater ICCs were almost perfect when measuring PiSca and MRI, and substantial to almost perfect for RaUl. Pearson‘s correlation showed a moderate, positive correlation between PiSca and RaUl distances (*r* = 0.608; *p* < 0.001), and a moderate, negative correlation between RaUl distance and MRI shift (*r* = − 0.486; *p* = 0.010). When the three core groups (peripheral, central tear, controls) were compared to each other regarding the radiographic instability parameters, only the MRI shift revealed a statistically significant difference (*p* = 0.003). Comparisons revealed significant differences between patients and controls (*p* = 0.004) and peripheral tears vs. controls (*p* = 0.001 and *p* = 0.010). The ROC analysis revealed a significant AUC only for the MRI (AuC 0.787 and *p* = 0.002).

**Conclusions:**

Children and adolescents with peripheral TFCC tears showed significantly increased instability parameters in MRI compared to controls. These measurement techniques are no replacement for a thorough clinical examination but may be helpful for indicating diagnostic wrist arthroscopy in ambiguous cases.

**Level of evidence:**

Level III; Diagnostic.

## Introduction

The aetiology of pain and discomfort in the distal radioulnar joint (DRUJ) is various and may be unspecific, making it difficult to diagnose by clinical means [1–3]. Commonly, young patients report a history of recent or past wrist injury and present with symptoms such as ulnar-sided wrist pain, clicking phenomenons in the DRUJ, piano key sign, decreased grip strength and subjective instability. Often, patients suffered a fracture of the distal forearm and developed permanent pain and discomfort in the wrist joint [4]. However, there is an increasing number of reported cases without any fractures but isolated ligamentous injuries which subsequently led to DRUJ instability [5].

The triangular fibrocartilage complex (TFCC) is the main stabilizing structure of the DRUJ [6]. The detrimental effect of a concomitant DRUJ instability can be explained by looking at the TFCC anatomy in more detail. The stabilizing function of the TFCC on the DRUJ is provided by the dorsal and palmar radioulnar ligaments. The most important structure for stabilization consists of the deep portion of the TFCC and its foveal insertion. This part is also known as ligamentum subcruentum [6–8]. Hence, there might be a difference in peripheral and central tears and their effect on DRUJ integrity [9]. Thus, traumatic TFCC tears and neglected concomitant DRUJ instability may be predisposing for the development of chronic wrist pain. Damage to these structures and pain as a consequence thereof may be resolved by surgical repair of TFCC tears [10–14]. Although it has been commonly assumed that DRUJ instabilities due to sole ligamentous injuries are rather rare in children, recent studies reported different findings [5]. TFCC tears occur more frequently after radial and/or ulnar fractures with epiphyseal lesions which potentially lead to posttraumatic radioulnar growth differences, while isolated TFCC tears are mostly caused by direct axial trauma to the forearm [4, 15]. This poses another problem after growth cessation as individuals with an ulnar variance may be more prone to TFCC wear and lesions [16].

The clinical presentation of the patient may be difficult to interpret due to the complex anatomy of the human wrist. Hence, recent reports suggest the use of standardized measurement techniques on patient’s radiographs, computed tomography (CT) and magnetic resonance imaging (MRI) as an additional diagnostic tool [17–22]. The aim of this study was to evaluate the efficacy and accuracy of (1) the MRI-based modified radioulnar ratio technique [17, 23] and (2) the pisoscaphoid (PiSca) and radioulnar (RaUl) distance on lateral radiographs [24] in children with arthroscopically-verified TFCC tears. We aimed to determine predictive factors for the presence of acquired TFCC tears.

## Materials and methods

### Case and control group

This retrospective case–control study (diagnostic; level III) aimed to assess the diagnostic validity and reliability of (1) the modified radioulnar ratio measured on MRIs, and (2) the pisoscaphoid- and radioulnar distances assessed in true lateral wrist radiographs. For this reason, we first queried our hospital database for patients who had received wrist arthroscopy at our tertiary referral center. The inclusion criteria were as follows: age under 19 years at the date of surgery, arthroscopically verified and classified TFCC tear according to Palmer [22, 25], preoperative presence of a lateral wrist radiograph and an MRI of the affected wrist. The Palmer classification briefly distinguishes between traumatic (type 1) and degenerative tears (type 2). Moreover, tears can be located in the central- (1A) or radial-sided (1D) portion of the TFCC or at the peripheral margin/capsule (1B). The radiographs were checked for true lateral radiograph criteria according to Mino et al. [18] Radiographs were taken with the patient seated with an upright torso. The humerus was positioned in 90° abduction and the forearm in a neutral position. The radiograph source was centered perpendicular to the carpus with the radiograph plate placed underneath it. A complete superimposition of the lunate, proximal pole of the scaphoid, and triquetrum was achieved, the radial styloid was centered over the proximal carpal row [18]. Exclusion criteria were: age > 19 years, incomplete patient history, suboptimal image quality or positioning or concomitant wrist pathologies. Standardized wrist radiographs were routinely made at our hospital preoperatively, MRIs were obtained by the patients in an extramural setting. Contrast materials were not used routinely. For the MRI images, patients were positioned in a prone position with a 180° elevated arm and a pronated forearm.

The control group consisted of adolescent cases who had routine imaging obtained for various reasons such as preoperative side comparisons or other minor hand/finger pathologies not affecting wrist stability. The inclusion criteria were eventually met by 18 patients (22 wrists); 14 females and four males with a mean age of 15.7 years (SD ± 2.9) at the date of surgery. These children and adolescents were compared to a control group of 28 patients (28 wrists; eight males, 20 females) with a mean age of 15.7 years (SD ± 3.3). Surgery was performed by two highly-experienced pediatric hand specialists (level IV according to Tang and Giddins) [26]. Detailed patient data can be found in Table 1.

**Table 1:** Demographic details

Patient	Wrist	Sex	Side	Age at surgery (years)	Palmer classification
1	1	M	R	16	1B
	2		R (re-tear)	18	2A
2	3	F	L	18	2C
	4		R	18	1A
3	5	M	L	17	1B
4	6	F	L	13	1B
5	7	F	R	17	2A
6	8	F	L	14	1B
7	9	F	L	18	1A
	10		R	18	1D
8	11	F	R	10	1B
9	12	M	R	14	1B/D
10	13	F	L	9	1B
11	14	F	L	16	1A
	15		R	16	1B
12	16	M	L	14	1A
13	17	F	R	18	1A
14	18	F	R	17	1D
15	19	F	L	18	1B
16	20	F	R	14	1B
17	21	F	L	14	1B
18	22	F	L	11	2A

*M* male, *F* female, *L* left, *R* right

### Measurement technique

A multiple examiner setting was used to conduct this study. We aimed to test for accuracy and clinical feasibility of the modified radioulnar ratio method [17, 23, 27] and the RaUl and PiSca distances [28]. Therefore, three examiners with different medical experience (medical student, surgeon-in-training, board-certified surgeon) familiarized themselves with the measurement technique. After completing a pilot session of ten cases, each observer measured each wrist in two independent sessions two weeks apart. The measurers were blinded against each other and against cases vs. controls. Syngo Studio version VB36E (Siemens Healthcare GmbH, Erlangen, Germany) was used for measuring purposes.

**Fig. 1:**
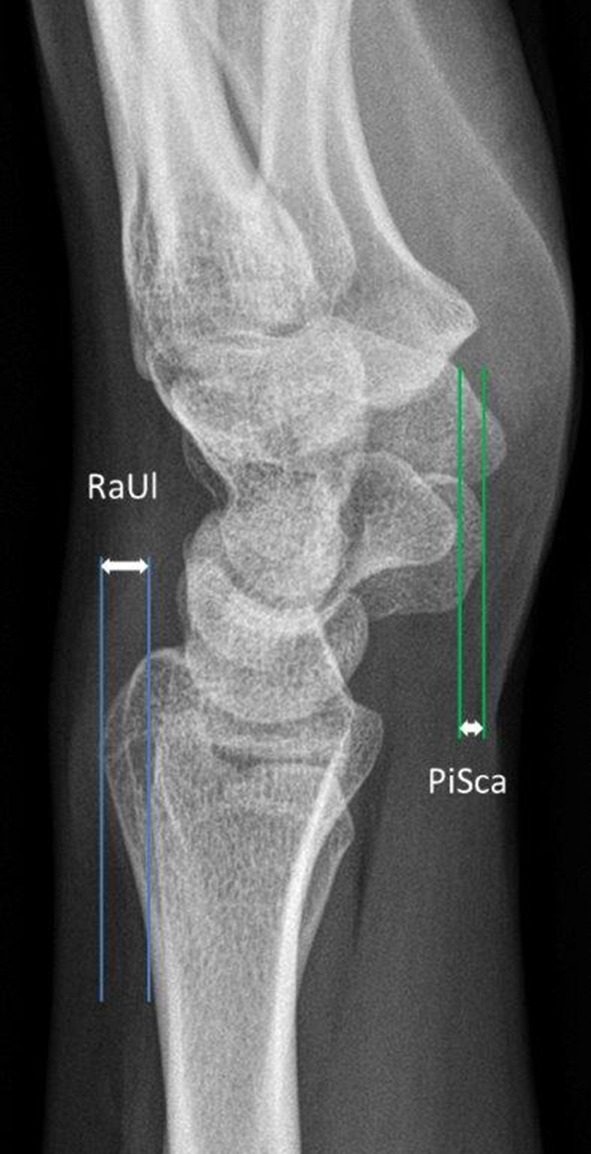
Schematic drawing of the RaUl and PiSca distance

**Fig. 2:**
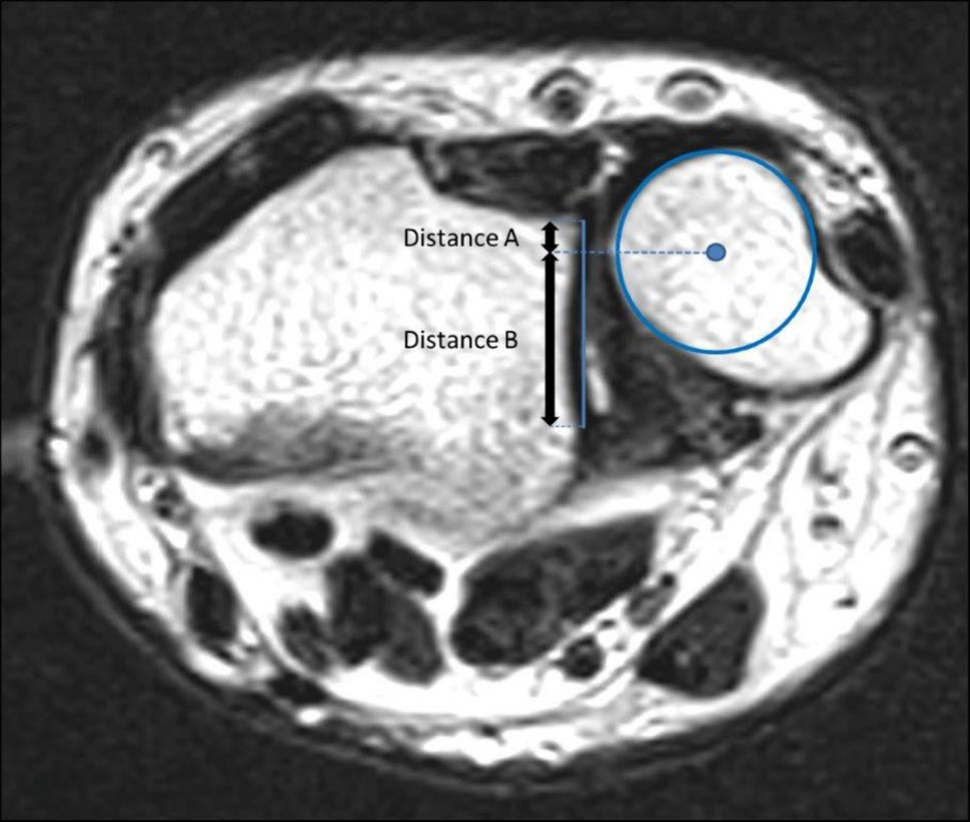
Schematic drawing of the modified radioulnar ratio

True lateral radiographs of the affected wrist joints were measured as described by Nakamura et al. [28]. The dorsal aspect of the ulnar head and Lister’s tubercle were identified. An auxiliary line was drawn tangentially to each structure. The distance between both was measured to determine the RaUl in millimeters (negative value = ulna dorsal to radius; Fig. 1). The same technique was used to measure the distance between the palmar tubercle of the scaphoid and the pisiform which determines the PiSca distance (negative distance = scaphoid is palmar to the pisiform; Fig. 1). Axial MRI sequences with a clearly depicted sigmoid notch, the most distal aspect of the ulnar head and a well visible Lister’s tubercle were mandatory for measurement purposes according to the recommendations of Ehman et al. [17]. First, the distance of the sigmoid notch was measured from the dorsal to the palmar aspect. Next, the axial center of the ulnar head was determined and an auxiliary line perpendicular to the line spanning the sigmoid notch was drawn. Then, the palmar and dorsal segment of the line were measured to quantify the ulnar displacement (Fig. 2). This was achieved by using the formula previously published by Ehman et al. *B*/(*A* + *B*) × 100% − 50% [14]. Thus, positive values were seen in dorsal displacement and negative values in the palmar displacement of the ulnar head.

### Statistical analysis

Intraclass correlation coefficients (ICCs) were calculated to evaluate the agreement between the three observers and within the same observer at different time points. The two-way mixed model (single measures) with absolute agreement was used, and confidence intervals (95% CI) are reported. To interpret the overall agreement, we adhered to the criteria published by Landis and Koch, where 0 indicates no agreement, 0.01–0.20 slight, 0.21–0.40 fair, 0.41–0.60 moderate, 0.61–0.80 substantial, and 0.81–1.0 almost perfect agreement [29]. Due to the determined high grade of intra- and interrater reliability found during the analysis, further calculations were performed using the mean measurements of the six values obtained by the observers for each outcome variable (PiSca, RaUl, MRI). Pearson’s correlation was performed to establish the correlation between the variables. Then, unifactorial ANOVA was performed to compare the groups (TFCC tear peripheral/central; control group) with regards to the different radiographic variables. Since some patients had two measurements, these calculations were repeated with a mixed ANOVA model. Finally, a receiver operating characteristics (ROC) analysis with 95% CI calculation for diagnostic value according to Wilson was performed. In addition, sensitivity, specificity and accuracy were calculated.

## Results

Concerning the agreement of the measurements, the interrater ICCs were almost perfect for all measurements except RaUl1, which showed a substantial agreement (0.751) among the three observers (Table 2). As expected, the second measurement series provided slightly superior results compared to the first one. The intrarater ICCs were almost perfect for all three raters when measuring PiSca and MRI shift. For RaUl, the ICCs were almost perfect for raters 2 and 3, and substantial for rater 1.

**Table 2:** Inter- and intrarateragreement among the observers

Interrateragreement	Intraclass correlation	95% Confidence Interval	
		Lower bound	Upper bound
PiSca 1	0.91	0.84	0.95
RaUl 1	0.75	0.61	0.86
MRI 1	0.84	0.75	0.91
PiSca 2	0.94	0.89	0.97
RaUl 2	0.82	0.70	0.90
MRI 2	0.86	0.77	0.92
Intrarateragreement PiSca*			
Rater 1	0.83	0.68	0.91
Rater 2	0.92	0.84	0.96
Rater 3	0.99	0.99	0.99
Intrarateragreement RaUl*			
Rater 1	0.74	0.54	0.86
Rater 2	0.85	0.72	0.92
Rater 3	0.98	0.97	0.99
Intrarateragreement MRI*			
Rater 1	0.94	0.89	0.97
Rater 2	0.92	0.85	0.96
Rater 3	0.96	0.93	0.98

*PiSca* pisoscaphoid distance, *RaUl* radioulnar distance, *MRI* magnetic resonance imaging

*Rater 1, surgeon-in-training; rater 2, medical student; rater 3, board-certified surgeon

Pearson’s correlation showed a moderate, positive correlation between PiSca and RaUl distances (*r* = 0.608; *p* < 0.001), and a moderate, negative correlation between RaUl distance and MRI shift (*r* = − 0.486; *p* = 0.010); however, no correlation was observed between PiSca and MRI (*r* = 0.011; *p* = 0.956).

When the three core groups (peripheral tears [Palmer 1B] and central tears [Palmer 1A/2A], controls) were compared to each other regarding the radiographic instability parameters, only the MRI shift revealed a statistically significant difference (*p* = 0.003; Table 3). More specifically, contrast tests and pairwise post hoc comparisons revealed significant differences between patients and controls (*p* = 0.004) and peripheral tears vs. controls (*p* = 0.001 and *p* = 0.010, respectively) but not between central tears and controls (*p* = 0.134 and *p* = 0.526). Pairwise comparison of peripheral vs. central tears was not significantly different (*p* = 0.675). Hence, children and adolescents with (peripheral) TFCC tears showed significantly increased instability parameters in MRI but not in lateral radiographs compared to controls.

**Table 3:** Groupwise comparisons of TFCC Tears vs. controls regarding the radiographic outcome parameters

Variables	*N*	Mean	SD	Variables	95% Confidence Interval for mean		*p* value*
					Lower bound	Upper bound	
PiSca	Peripheral tear	12	− 2.40	3.34	− 4.53	− 0.28	0.529 (0.411)
	Central tear	9	− 2.80	2.21	− 4.50	− 1.11	
	Controls	14	− 1.33	3.71	− 3.47	0.81	
	Total	35	− 2.08	3.23	− 3.19	− 0.97	
RaUl	Peripheral tear	12	0.16	2.28	− 1.28	1.61	0.230 (0.094)
	Central tear	9	− 0.50	2.27	− 2.24	1.25	
	Controls	14	1.38	3.05	− 0.38	3.14	
	Total	35	0.48	2.66	− 0.43	1.39	
MRI Shift	Peripheral tear	12	0.22	0.13	0.13	0.30	**0.003 (0.006)**
	Central tear	6	0.15	0.16	− 0.01	0.32	
	Controls	24	0.08	0.08	0.04	0.11	
	Total	42	0.13	0.12	0.09	0.17	

Significant result highlighted in bold

*PiSca* pisoscaphoid distance, *RaUl*radioulnar distance, *MRI* magnetic resonance imaging

*p value in bracket reflects the result of the mixed ANOVA model between tears and controls

**Fig. 3:**
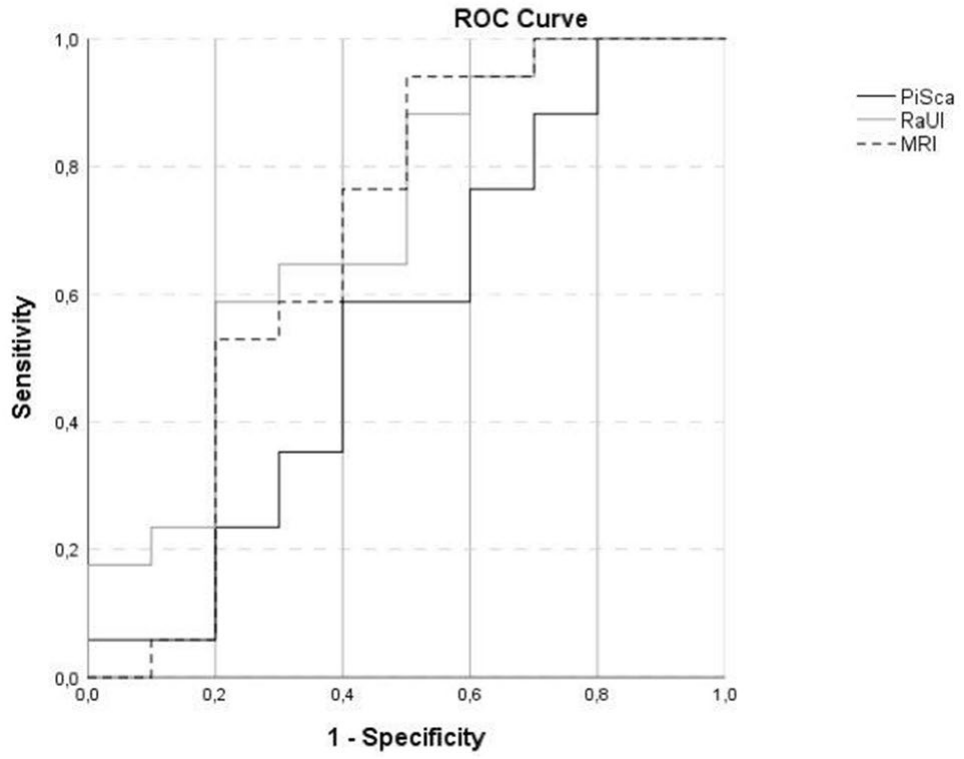
ROC curve for determining sensitivity and specificity

These results were corroborated in the ROC analysis, which revealed a significant “Area Under The Curve” only for the MRI (AuC 0.787 and *p* = 0.002) but not PiSca (AuC 0.599 and *p* = 0.329) and RaUl parameters (AuC 0.639 and *p* = 0.167). When using a cut-off value of 10.3% MRI shift, a sensitivity of 83.3% (95% CI 60.8%, 94.2%), a specificity of 66.7% (95% CI 46,7%, 82.0%), and an accuracy of 73.8% (95% CI 58,9%, 84.7%) could be achieved (Fig. 3).

## Discussion

DRUJ instabilities as sequelae of traumatic TFCC tears may be underdiagnosed in children and adolescents [4]. In general, this type of ligamentous injury was assumed to be very rare and often overlooked. This may be due to insufficient examiner experience, diagnostics or mild, unspecific symptoms. However, recent studies reported different findings. Thus, isolated TFCC tears with concomitant DRUJ instability must be taken into consideration in patients with ulnar-sided wrist pain [4, 30]. Early treatment may be important in symptomatic patients with traumatic tears with pain duration being essential for achieving satisfactory results [31].

Clinical examination is of paramount importance to assess the DRUJ and its stability at the first presentation of the patient. However, various measurement techniques have been published to substantiate the severity of any DRUJ pathology. Mino first reported the use of true lateral radiographs and wrist CTs to confirm the diagnosis of DRUJ instability in 1983 [18]. They hypothesized later that DRUJ incongruities may be diagnosed with a true lateral radiograph only [19]. However, we believe that this may be the case in severe subluxations or even dislocations but is difficult to determine in slight subluxations or mild, dynamic instability, which is often seen in patients with traumatic TFCC tears.

Lo et al. [20] reported the results of 100 measured CT scans and reported that this method was superior compared to the epicenter method and Mino criteria [18, 19] with regards to detection of DRUJ instability [23]. They concluded that this technique may be a sensitive method to detect cases with slight instability. We agree with this claim and confirmed it with our MRI-based data. Thus, we believe this method to be well-suited in a pediatric population such as presented in this study.

Ehman et al. reported on 34 patients with surgically verified foveal TFCC tears [17]. They adapted the techniques which were previously published by Lo et al. and Ishikawa et al. [21, 23]. A specificity of 91% and a sensitivity of 62% was obtained. According to their results, a subluxation of 11% is considered pathological. Therefore, we opted to use the same technique which focuses on the position of the ulnar head in relation to the sigmoid notch. Simultaneously, this technique offers a high feasibility and may be routinely used by medical professionals with different levels of hand surgical experience as shown by our satisfactory interrater reliability. Recent data shows that symptomatic patients may benefit from surgical debridement of the central disc [32]. Therefore, we also examined central tears in addition to foveal tears. According to our results with only a limited sample size of central tears, there are no distinct signs of radiological subluxation in patients with central tears. In summary, based on our results, the modified radioulnar ratio technique may be considered a useful additional diagnostic tool, but of course not a replacement for a thorough wrist examination.

Nakamura et al. [24] reported the results of 56 patients with ulnar-sided wrist pain after fractures or soft tissue injuries and evaluated the pisoscaphoid and radioulnar distances. The authors stated that a radioulnar difference of six millimeters or above in patients with less than three millimeters in pisoscaphoid difference between both the injured and non-injured wrist is considered a positive diagnostic finding for a DRUJ dislocation. The authors recommended to perform CT imaging for borderline cases with 4–5 mm of radioulnar difference. Our data obtained from the injured unilateral, true lateral wrist radiographs show that the absolute PiSca and RaUl distances may not be well-suited to detect radiological instabilities in a pediatric cohort. Thus, we cannot recommend the use of this technique without bilateral radiograph comparison to evaluate potential instability in patients with arthroscopically-verified TFCC tears.

According to recent data, the diagnosis of a torn TFCC may take up to 18 years [5]. MRI arthrography is considered superior as it grants better differentiation. However, it is considered an invasive examination which might not be suited for the use in children and adolescents. High-resolution 3 T MRIs availability is increasing and shows more detailed image quality. Despite showing promising results for ligamentous injuries in the wrist, the specificity concerning TFCC lesions is still unsatisfactory [33].

Our results have shown that instability signs such as palmar or dorsal shift of the ulnar head were significantly more often present in cases with TFCC tears (central and peripheral), and especially in those with peripheral tears. A cut-off value of around 10% of dislocation showed a reasonable sensitivity/specificity ratio. Thus, as mentioned above, this technique can be used preoperatively to determine potential TFCC tears in children and adolescents and may provide help for surgical indication setting in doubtful cases. In such cases, diagnostic wrist arthroscopy is still considered the diagnostic gold standard [19, 30].

This study has potential limitations. We acknowledge a rather small sample size as DRUJ instability after traumatic TFCC tears is still a rather rare aetiology in children and adolescents. We further recommend ambidextrous imaging to compare the affected to the healthy side as mild radiological subluxation might not be necessarily pathological. The case cohort in this study did not have bilateral wrist images uniformly available. Finally, we acknowledge a potential bias because of different radiology institutes where patients received their MRIs. However, we believe that this in turn has to be considered a strength of the study as it was conducted under realistic outpatient circumstances with a high overall quality and level of standardization in musculoskeletal imaging in our country. Finally, we acknowledge that there was a lack of precise documentation concerning clinical instability findings in our patient charts. Hence, we were not able to include clinical findings as a supportive variable in our analysis. Nevertheless, we can confirm that no case with severe instability was included as these cases underwent different surgical techniques to restore DRUJ stability.

Mild to moderate instability of the DRUJ is often somewhat difficult to diagnose with sole clinical examination in children and adolescents due to ligamentous laxity, and symptoms concerning the DRUJ and/or TFCC may be difficult to judge. Our results provide additional radiological insight and decision-making support whether DRUJ instability may be present and thus whether surgical exploration or intervention may be warranted.
